# Lipid-coated ZnO nanoparticles synthesis, characterization and cytotoxicity studies in cancer cell

**DOI:** 10.1186/s40580-020-00224-9

**Published:** 2020-04-23

**Authors:** Dingding Cao, Xugang Shu, Dandan Zhu, Shengli Liang, Murtaza Hasan, Sheng Gong

**Affiliations:** School of Chemistry and Chemical Engineering, Zhongkai University of Agriculture and Engineering, Guangzhou, 510220 China

**Keywords:** Zinc oxide, Nanocrystals synthesis, Lipid-coated ZnO, Cytotoxicity

## Abstract

ZnO nanoparticles are widely used in biological, chemical, and medical fields, but their toxicity impedes their wide application. In this study, pristine ZnO NPs (~ 7 nm; ~ 18 nm; ~ 49 nm) and lipid-coated ZnO NPs (~ 13 nm; ~ 22 nm; ~ 52 nm) with different morphologies were prepared by chemical method and characterized by TEM, XRD, HRTEM, FTIR, and DLS. Our results showed that the lipid-coated ZnO NPs (~ 13 nm; ~ 22 nm; ~ 52 nm) groups improved the colloidal stability, prevented the aggregation and dissolution of nanocrystal particles in the solution, inhibited the dissolution of ZnO NPs into Zn^2+^ cations, and reduced cytotoxicity more efficiently than the pristine ZnO NPs (~ 7 nm; ~ 18 nm; ~ 49 nm). Compared to the lipid-coated ZnO NPs, pristine ZnO NPs (~ 7 nm; ~ 18 nm; ~ 49 nm) could dose-dependently destroy the cells at low concentrations. At the same concentration, ZnO NPs (~ 7 nm) exhibited the highest cytotoxicity. These results could provide a basis for the toxicological study of the nanoparticles and direct future investigations for preventing strong aggregation, reducing the toxic effects of lipid-bilayer and promoting the uptake of nanoparticles by HeLa cells efficiently.

## Highlights

- Morphology of ZnO nanoparticles (NPs) can be controlled in ethanol.
- ZnO NPs surface could successfully self-assemble the phospholipid bilayer.
- Phospholipid layer can avoids ZnO NPs dissolution into Zn^2+^ cations.
- At the same concentration, small particle size ZnO NPs has the higher cytotoxicity.
- Lipid-coated ZnO NPs shows low cytotoxicity.

## Introduction

Zinc oxide (ZnO) is extensively used in physical chemistry, biomedical sciences, catalysts, transducers, microelectronics, textile, cosmetics, and other applications, because of its small particle size and high specific surface area [1–3]. However, the size, shape, aspect ratio, specific surface area, and surface chemistry should be maintained at the desired levels for the chemical and biomedical applications of ZnO nanoparticles [4, 5].

Because of the large specific surface area, small size effect, and other physical and chemical properties, the ZnO nanocrystals exhibit different toxicity under different environments. Well-dispersed nanoparticles are more toxic than poorly dispersed ones [6]. As the nanomaterials are prone to agglomeration [7], the preparation of stable and well-dispersed ZnO nanocrystal in aqueous media is quite challenging. Practically, the size and surface properties of the ZnO nanoparticles largely determine the extent of agglomeration [8]. The cytotoxicity of ZnO nanomaterials is related to the surface nanostructure and the extent to be internalized by cells [9]. As the surface properties and shape of the ZnO nanocrystals significantly influence the cytotoxicity, these parameters should be maintained at the desired levels [10, 11].

In previous researches, different methods were followed to decrease agglomerations, and improve dispersion, stability, and biocompatibility of the nanomaterials [12, 13], such as an organic coating or addition of a capping agent (polyethylene glycol (PEG), l-dopa, poly-n-vinyl-2-pyrrolidone (PVP) and others [14, 15]. However, the cytotoxic effects of capping agents or coatings have also been reported [16]. Herein, we prepared ZnO NPs, coated with the lipid bilayer and characterized their properties.

Phospholipids, cholesterol, and proteins are the main building blocks of cell membranes. Phospholipids maintain the dynamic and structural functions of cell membranes [17]. They have three major components: a phosphate head group (hydrophilic or polar), the glycerol backbone and two fatty acid tails (long carbon chains, hydrophobic). In water, the hydrophilic heads remain close to the water, while the tails orient themselves away from that. Then, these phosphate groups cluster together and form phospholipid bilayers (lipid bilayer) [18]. Cholesterol is very important for the cell membrane. Cholesterol has a pretty stable structure; it can randomly insert itself between the phospholipids, and help maintain the fluidity of the cell membranes. Therefore, the interactions between phospholipids and potential cell-membrane damaging agents should be studied for understanding the biological effects [19].

The isoelectric point of ZnO nanoparticles is generally about pH 9–10, and they are positively surface charged in aqueous solution. The surfaces of pristine ZnO NPs contain many neutral hydroxyl groups, which govern the surface charge behavior of nanomaterials [20]. The stability of ZnO nanocrystal colloids is also affected by charge and surface chemistry, which are important for the interaction between inorganic nanoparticles and organic organisms [21]. HeLa cells usually possess a high negative membrane potential due to anionic phospholipids or other charged proteins and other active substances. So, the positively charged ZnO nanocrystals bind to anionic phospholipids through electrostatic interactions, which in turn affect the cancer cells [22–24].

In recent years, many studies have been focused on the toxicological effects of ZnO NPs [25–27]. More specific data, such as morphology and chemical modification, should be compiled for predicting the cytotoxicity of the nanoparticles [28, 29]. Only a few studies have compared the toxicities of ZnO NPs of different morphologies, and even fewer toxicity studies have been published on lipid-coated ZnO NPs [30, 31].

Coarse nanoparticles can be inhaled by the lungs into the systemic circulation. Coarse particles are mainly deposited in the upper respiratory tract, fine particles are inhaled deep into the lungs, leading to pneumonia; so, different morphologies of nanocrystals can induce physiological inflammation [32]. Furthermore, biological assays can be employed for studying the toxicity of nanomaterials, for example, the assays for cytotoxicity and apoptosis [33].

According to some reports, nanoparticles release their ions under the physiological conditions, and the number of ions released by the nanoparticles with different morphologies is different. The dissolution of nanocrystals triggers the release of zinc ions, and reactive oxygen species (ROS) are generated because of the internalization of nanocrystals [34]. According to some reports, cytotoxicity does not depend upon the extracellular soluble Zn^2+^ concentration, while the direct contact or internalization of ZnO nanocrystals with HeLa cells may lead to higher cytotoxicity [35, 36]. Other studies have shown that Zn^2+^ cations can trigger the formation of intracellular ROS, which may be the main factor for oxidative stress and cell damage [37]. Furthermore, the NF-nB isoform is upregulated in response to diverse stimuli. The local concentration effect and oxidative stress may also cause cell damage [38].

Based on the above considerations, the preparation and surface functionalization of ZnO NPs with different morphologies have been investigated for improving the biological stability and biocompatibility, and a systematic approach has been proposed for studying the stability and cytotoxicity. For each morphology of ZnO (~ 7 nm, ~ 18 nm, ~ 49 nm) and lipid-coated NPs (~ 13 nm, ~ 22 nm, ~ 52 nm), the cytotoxicity was evaluated. We attempted to provide a basic set of data to determine the difference in cytotoxicity.

In the study, ZnO NPs of different morphologies were prepared by the wet chemical method. Six types of ZnO nanoparticles were designed: (i) pristine ZnO NPs, which were rich in hydroxyl groups (~ 7 nm; ~ 18 nm; ~ 49 nm), and (ii) lipid-coated ZnO NPs (~ 13 nm; ~ 22 nm; ~ 52 nm). The physicochemical properties of the samples were characterized. We studied the stability of these samples in aqueous solution. The effects of ZnO nanocrystal’s surface properties on cellular internalization and cytotoxicity were studied.

In detail, we exhibited that lipid-coated ZnO NPs can greatly improve the colloidal and chemical stability in aqueous solution. Furthermore, we utilized HeLa cells as the target cells. After 24 h of exposure in the ZnO NPs (~ 7 nm, ~ 18 nm, ~ 49 nm) and lipid-coated ZnO NPs (~ 13 nm, ~ 22 nm, ~ 52 nm), the cells were observed under a biological transmission electron microscope, and the effect of ZnO NPs on the cellular morphology was studied. We further investigated the cytotoxic mechanisms of ZnO and lipid-coated NPs with different morphologies and provided corresponding evidence and conclusions. These results can provide basic data regarding the biological effects of ZnO with different morphologies of exposure to cells, which can be used to understand the toxicity of the nanocrystal in the biological matrix. The results of our study can guide future researches on the cytotoxicity of ZnO nanocrystals with different morphologies, especially the biological effects of organic coatings on the interactions of inorganic materials.

## Materials and methods

### Materials

Zinc acetate dihydrate (Zn(O_2_CCH_3_)_2_(H_2_O)_2_), sodium hydroxide (NaOH) and 1,2-Diacyl-sn-glycerol-3-phosphocholine (DOPC) were purchased from Shanghai Aladdin Biochemical Technology Co. (China). Absolute ethanol was obtained from Tianjin Damao Chemical Reagents Co. (China). Cholesterol was purchased from A.V.T (Shanghai) Pharmaceutical Co., Ltd. PBS (pH 7.2–7.4) was purchased from Beijing Solarbio Science & Technology Co., Ltd. All reagents were analytically pure and used as received without any further purification.

### Synthesis of ZnO nanoparticles with different morphologies

ZnO NPs were prepared at standard conditions: reaction temperature of 60 °C; 2.25, 12 h or 24 h of reaction time, and 7.22 and 3.73 mmol of NaOH and zinc acetate dihydrate, respectively, as the starting materials. Final products were obtained as white precipitates. As discussed elsewhere [39, 40], the synthetic mixture was prepared from two different solutions: solution A and B; solution A contained 3.73 mmol of zinc acetate dihydrate dissolved in 40 mL of ethanol; solution B contained 7.22 mmol of NaOH dissolved in 320 µL of bi-distilled water and then in 25 mL of ethanol. Solution B was added dropwise to solution A under vigorous and constant stirring for 2.25, 12 h or 24 h at 60 °C, after which the solution was allowed to cool down to room temperature. The synthesized ZnO samples were collected by centrifuging, and then washed thoroughly with pure ethanol. This procedure was repeated several times. ZnO NPs were re-dispersed in ethanol or dried at 60 °C for 2 h. Finally, three morphologies of ZnO NPs (~ 7 nm, ~ 18 nm, ~ 49 nm) are obtained. All ZnO NPs were stored at room temperature. These samples were marked as ZnO NPs(~ 7 nm), ZnO NPs(~ 18 nm), and ZnO NPs(~ 49 nm). Please see Scheme 2a for the synthesis process. During the formation of NPs, (Zn(O_2_CCH_3_)_2_(H_2_O)_2_) reacted with NaOH in ethanol. The dehydrating properties of ethanol prevented the formation of zinc hydroxide [41].

### Preparation of lipid-coated ZnO nanocrystals with different sizes

**Scheme 1:**
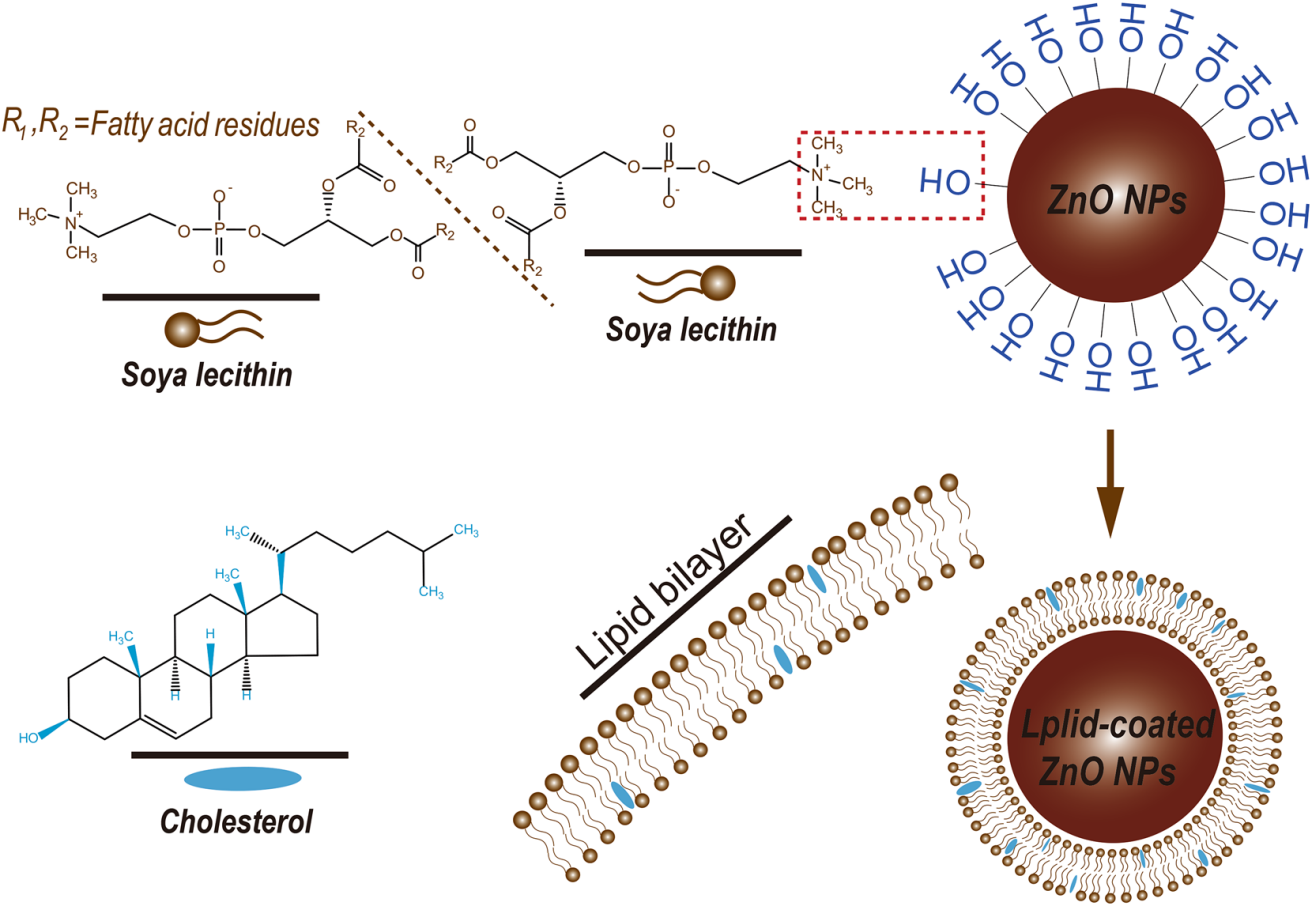
The schematic illustration of the synthesis of ZnO NPs and lipid-coated ZnO NPs

Similarly, the reaction mixture was also prepared from two different solutions: solution A and solution B; solution A contained 1.2 g of phospholipid, 0.08 g of cholesterol, and 0.08 g of ZnO NPs dissolved in 30 mL of ethanol; solution B contained 40 µL of Tween-80 dissolved in 20 mL of PBS (pH 7.2–7.4). Solution A was added dropwise to solution B under vigorous and constant stirring for 2 h at 60 °C. The mixture was then transferred to a rotary evaporator (110 rpm, 60 °C) for completely separating the ethanol. The samples were finally washed three times with PBS to remove free phospholipids, and dried in a vacuum freeze dryer. These samples were marked as lipid-coated ZnO NPs (~ 13 nm), lipid-coated ZnO NPs (~ 22 nm), lipid-coated ZnO NPs (~ 52 nm). Please see Scheme 1 for the synthesis process.

### Bio-stability assays (ICP-OES)

The bio-stability assays were performed at a concentration of 1 mg mL^−1^, by suspending 20 mg of ZnO NPs (~ 7 nm, ~ 18 nm, ~ 49 nm) and lipid-coated ZnO NPs (~ 13 nm, ~ 22 nm, ~ 52 nm) samples in 20 mL water. The samples were maintained under continuous stirring (110 rpm) at a constant temperature of 37 °C. At selected times (2, 12, 24, 48, 96 h), 3.5 mL of the suspension was collected and centrifuged. The supernatant was collected and diluted for ICP-OES analysis.

### Physico-chemical characterization

The morphologies of the samples were observed under an FEI Tecnai G2 F20 high-resolution transmission electron microscope (HR-TEM) coupled with energy dispersive X-Ray spectroscopy (EDX) and selected area electron diffraction (SAED) (Thermo Fisher Scientific, Waltham, MA, USA) following a standard procedure for negative staining. The cell-samples were characterized under a Hitachi H-7650 biological transmission electron microscope (Hitachi Limited, Japan). The crystal structures were detected using Smartlab X-ray powder diffractometer (XRD) with Cu Kα radiation (λ = 1.5418 Å) at the 2θ = 10–90° range and 5°/min of scanning speed. The hydrodynamic diameter (Dh) of the nanoparticles were characterized by dynamic light scattering (DLS) particle size analyzer (ELSZ-2, Otsuka Electronics Co., Osaka, Japan). ZnO NP suspensions were carefully sonicated before each experiment to minimize the aggregation effect. A TGA 2—thermogravimetric analyzer (Mettler-Toledo Columbus, OH, USA) was used to analyze the heat stability of these samples over the heating range of 40–800 °C and heating rate of 10 °C/min.

### Cell cultures

The HeLa cells lines were obtained from the Cell Bank of the R&S Biotechnology Co., Ltd (Shanghai, China). All cells were cultured in Dulbecco’s Modified Eagle’s medium (DMEM), (Keygen Biotech Co., Ltd., Nanjing, China) supplemented with 10% fetal bovine serum (10% FBS), 1% Glutamine (Gln) and 1% Penicillin–streptomycin (1% P/S), (Gemini, USA). The culture was maintained in a humidified incubator at 37 °C with 5% CO_2_ [42–44].

### Cell proliferation assay

Cell proliferation assay was conducted following the colorimetric water-soluble tetrazolium salt (CCK-8) assay using a Cell Counting Kit-8 (CK04, Dojindo, Japan) according to the manufacturer’s instructions. The cells were treated with different concentrations of ZnO and lipid-coated ZnO samples and incubated for 24 h at 37 °C. Thereafter, HeLa cells were fixed onto 96-well plates (8 × 103 cells/well). The cells were washed with PBS, and 10 μl of CCK-8 solution was added to each well and incubated for another 2 h at 37 °C. The number of the viable cells was determined by measuring the absorbance at 450 nm using a microplate reader (MuLTiSKAN-MK3, Thermo, USA). The cellular morphology was observed under an inverted fluorescence microscope (DMi8, Leica, Germany). In this experiment, the concentrations of the samples were distributed systematically from 15 to 200 µg/mL.

### Annexin V-FITC/propidium iodide (PI) apoptosis assay

Apoptosis of HeLa cells, induced by different samples was measured using a BD FASAria Cell Sorter (Beckton Dickinson, San Jose, CA, USA) with Annexin V-FITC/PI double staining method according to the manufacturer’s instructions. Briefly, the cells were harvested after 24 h of exposure to ZnO (7 nm) 40 µg/mL, ZnO (18 nm) 120 µg/mL, ZnO (49 nm) 150 µg/mL, lipid-coated-ZnO (49 nm) 40 µg/mL, lipid-coated-ZnO (49 nm) 120 µg/mL and lipid-coated-ZnO (49 nm) 150 µg/mL. Then, washed twice with cold phosphate buffer solution (PBS) and resuspended on the binding buffer. Then, annexin V-647-PI apoptosis detection Kit (Yeasen Biotech Co., Ltd., Shanghai, China) was added and mixed uniformly. Next, at least 10,000 cells were collected and detected by flow cytometry, and the percentages of apoptotic cells were analyzed by FlowJo V10 software.

### Statistical analysis

All cellular experiments were performed three times, and all the data were presented as the mean ± SD standard errors (SE). Multiple group comparisons of the means were performed by two-way analysis of variance (ANOVA) using GraphPad Prism version 7 (GraphPad Software, San Diego, CA). The difference between the means was considered statistically significant at *p < 0.05.

## Results and discussion

In this study, the wet chemical method was utilized for preparing the well-dispersed ZnO nanocrystals with three different morphologies, i.e. (~ 7 nm, ~ 18 nm, ~ 49 nm). Additionally, pristine ZnO nanoparticles were encapsulated with phospholipids (~ 13 nm, ~ 22 nm, ~ 52 nm) and their toxicological relationships were systematically explained. The lipid-coated ZnO NPs were prepared by functionalizing the pristine ZnO NPs and characterized by Fourier transform infrared (FT-IR), X-ray diffraction (XRD) and other characterization methods. The ability of the phospholipid bilayer to coat the nanoparticles relies on its self-assembly behavior. The basic principles of self-assembly are as follows: the lipid molecules can be dissolved in ethanol as monomers, containing a hydrophilic head and a hydrophobic tail. When water is added to the solution so that the monomers can self-assemble into liposomes composed of the lipid bilayer.

### Microscopic characterization of ZnO NPs

**Fig. 1:**
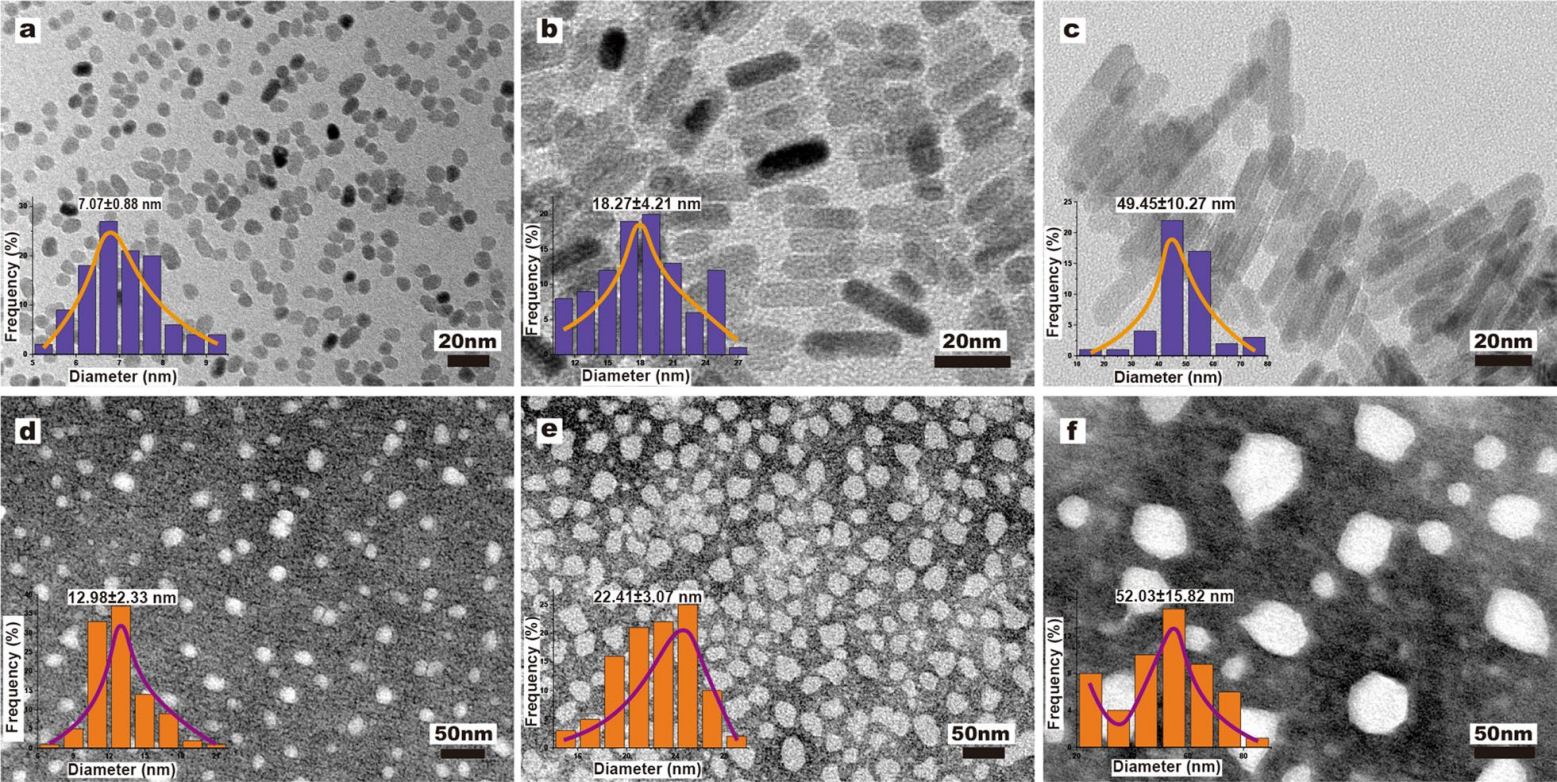
The characterization of samples. **a**–**c** The TEM images and size distribution of pristine ZnO NPs (~ 7 nm; ~ 18 nm; ~ 49 nm). **d**–**f** Lipid-coated ZnO NPs (~ 13 nm; ~ 22 nm; ~ 52 nm), these samples were subjected to the negative staining techniques. In panels (**a**–**f**) the inserted figures show the size distribution of samples from the TEM images, n = 100 or 50

The micro-characterization of the samples was performed through TEM analysis. The result revealed that these samples have different morphologies as shown in Fig. 1 (pristine ZnO NPs and their corresponding phospholipid encapsulated forms). The average diameter of samples is expressed as mean size ± SD nm. The average particle sizes of pristine ZnO NPs and lipid-coated ZnO NPs were: 7.07 ± 0.88 nm, 18.27 ± 4.21 nm, 49.45 ± 10.27 nm, 12.98 ± 2.33 nm, 22.41 ± 3.07 nm, and 52.03 ± 15.82 nm, respectively (Fig. 1, as measured from TEM images, n = 100 or 50). In frequency analysis, the samples displayed a narrow distribution peak, which indicated that the samples were well distributed.

**Fig. 2:**
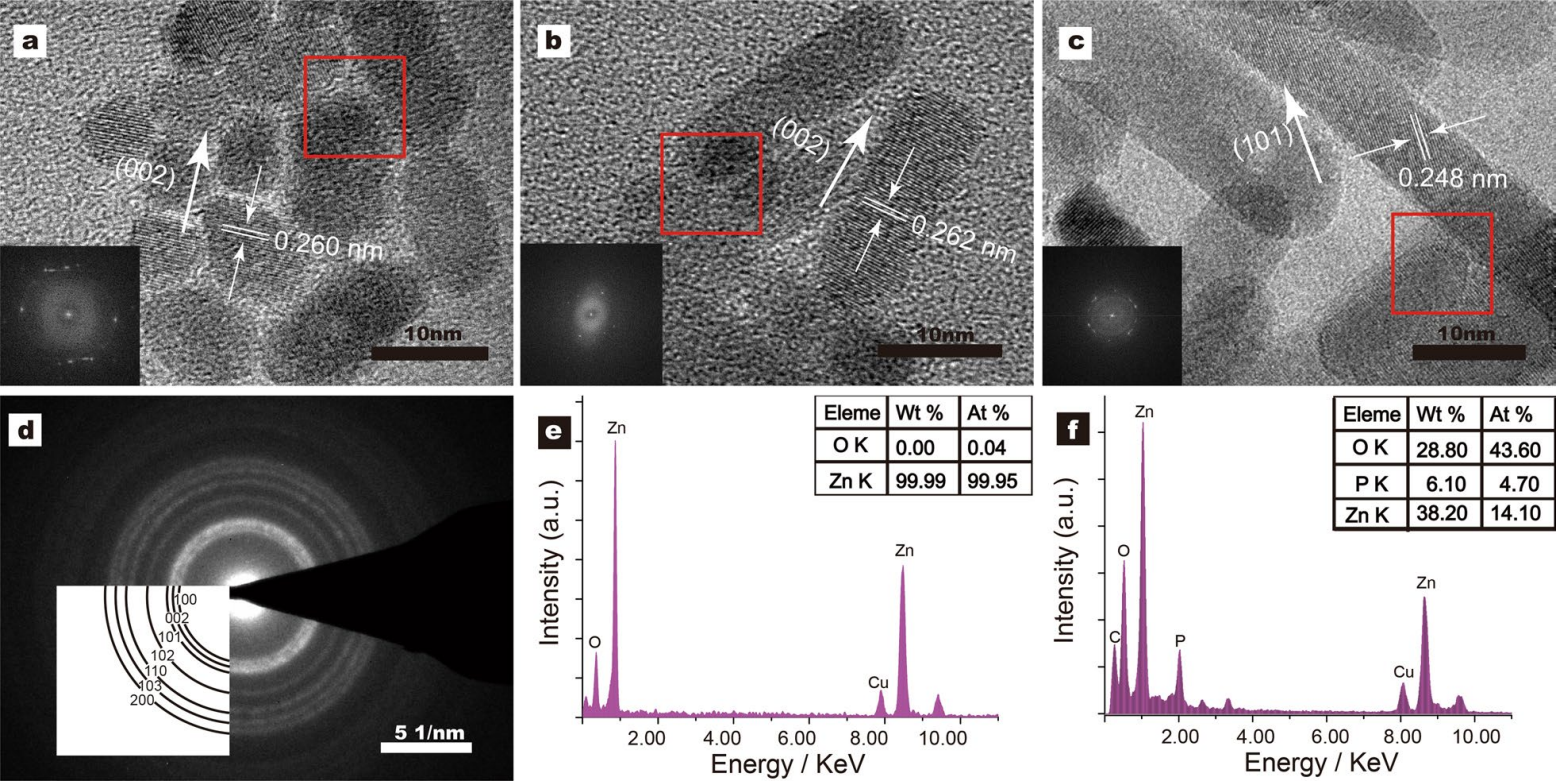
The characterization of ZnO NPs structure (**a**–**c**). The HRTEM images of Pristine ZnO NPs (~ 7 nm; ~ 18 nm; ~ 49 nm). The inserted photos show the Fourier filtered image from the red box. **d** The SAED pattern shows a wurtzite pattern from sample ZnO NPs (~ 7 nm). **e** The EDX spectrum results from sample ZnO NPs (~ 7 nm), and **f** Lipid-coated ZnO NPs (~ 13 nm)

The high-resolution TEM images (Fig. 2a–c) show that these three-particle morphologies all have high distinguishable morphology and well dispersion. All the sample was in the crystalline state. The HRTEM study suggests that the orientation of the crystal plane is not the same (Fig. 2a–c). The fast Fourier transform (FFT) patterns (Fig. 2a–c) demonstrate a single crystal hexagonal structure with 2.60 Å, 2.62 Å or 2.48 Å spacing between two adjacent lattice fringes, corresponding to (002) or (101) planes of wurtzite [45]. The SAED pattern also shows a wurtzite pattern for sample ZnO NPs (~ 7 nm) (Fig. 2d). A strong diffraction peak (002) of ZnO (Fig. 5a–f) is consistent with the ZnO NPs shape observed by TEM. XRD and HRTEM results confirmed that the preferential growth direction for ZnO NPs was oriented arrays was along the C-axis (see Fig. 2a–c). Each particle in the nanocrystal is a single crystal, and the HRTEM image displays the crystal surface. In Fig. 1, FFT suggests that their overall appearance is consistent, with the prolonged reaction time; the particle size may increase according to classical crystal models (Ostwald ripening) and oriented attachment (OA) principle and eventually become ~ 50 nm long and ~ 10 nm wide [46].

After functionalization with a lipid layer (Fig. 1d–f), the crystal surface structure of ZnO NPs did not modify (Fig. 5). The lipid-coated ZnO NPs exhibited larger particles than pristine ZnO NPs. A possible reason is the formation of a supported phospholipid layer of a few nm in thickness on the surface of ZnO nanocrystals [47, 48]. Besides, due to the presence of a phospholipid layer on the surface of ZnO nanocrystal, HR-TEM images did not show higher multiples. Although the lattice orientation was not observed, the overall distribution and aggregation of nanoparticles were assessed.

Next, the elements of the samples were detected by electron dispersive x-rays (EDS). Impurities might impact the cytotoxicity of nanoparticles. The EDX spectra (Fig. 2f and Additional file 1: Figure. S1) of lipid-coated ZnO NPs showed the presence of Zn (from ZnO NPs), and P (from phospholipid); this is consistent with the FT-IR results.

According to TEM morphological characteristics (Fig. 1) and EDX spectra (Fig. 2e–f and Additional file 1: Figure S1), the samples showed good crystallinity and no impurity. The EDS analysis of pristine ZnO NPs (~ 7 nm), showing 99.99% of Zn content, indicates high purity (Fig. 2e). The presence of the phospholipid layer in the ZnO nanocrystals surface was confirmed by the increase of phosphorus content in Lipid-coated ZnO NPs.

### Analysis of oriented attachment

The classical crystal growth models (Ostwald ripening) state that large nanoparticles grow at the cost of the small ones in a supersaturated reaction solution [49]. However, according to non-classical crystal growth models (oriented attachment OA), crystals grow by the repeated merging of particles on lattice-matched crystal facets [50–52]. In this section, we would examine the possibility of non-classical crystal growth in ZnO NPs’ synthesis and discuss the corresponding processes of NPs formed by OA.

**Fig. 3:**
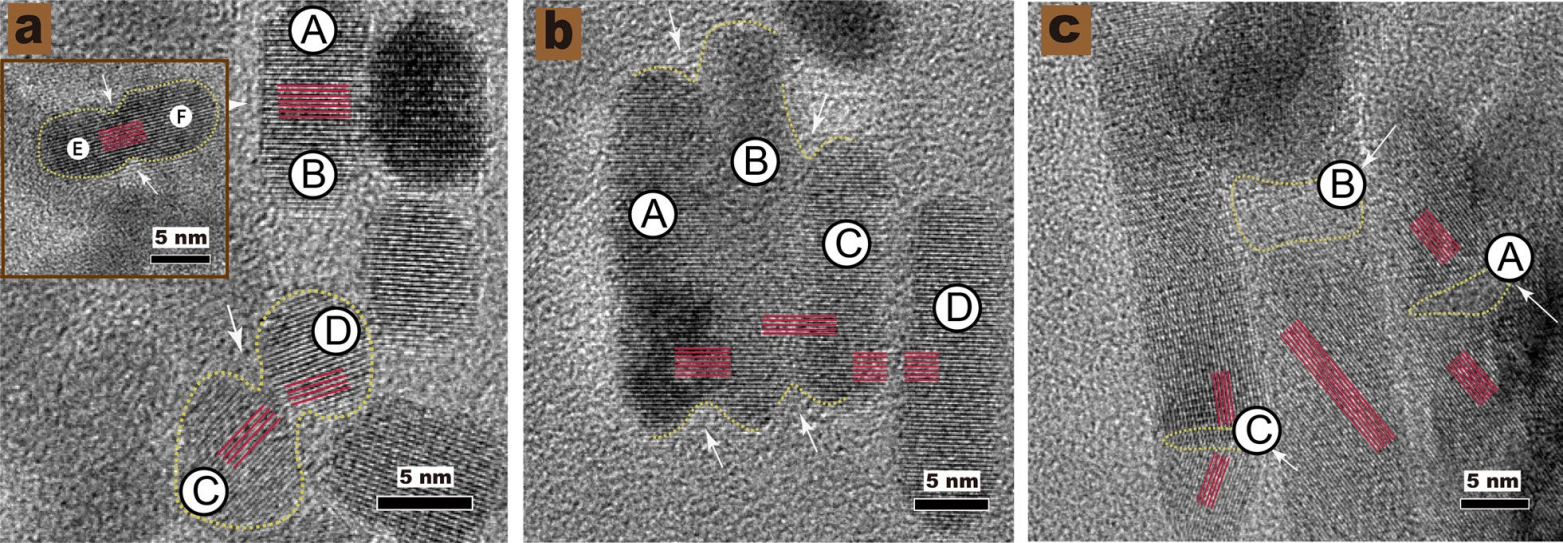
The HRTEM images of pristine ZnO NPs. **a** ZnO NPs (~ 7 nm); **b** ZnO NPs (~ 18 nm); **c** ZnO NPs (~ 49 nm). The ZnO NPs formed layer-by-layer either parallelly or perpendicularly to the C-axis. The arrows indicate the boundaries of the pristine particles

In this experiment, the reaction system reduced the overall surface energy by matching the crystal lattices and diminishing exposed areas and defects [53, 54]. The NPs clustered together and grew. Figure 3a–c (HRTEM) shows that the adjacent particles merge to form large particles. Figure 3b shows that the areas A-B-C have “aligned” into each other and maintained their perfect relative crystallographic orientation. The lattice planes of the merged particles were almost perfectly aligned. The adjacent NPs were arranged either parallelly or perpendicularly (see Fig. 3a, areas E-F and A-B) like a wall [55], and resulted in the elongation of ZnO NPs’ along the c-axis. Besides, the misorientation angles were formed between blocks among areas C-D (see Fig. 3a), which is consistent with other reports. Except for the phenomenon of dislocations, the bottlenecks and poorly merged fragments between the aligned dimers were also observed (see Fig. 3a, areas C-D and Fig. 3c, areas C and A). Yet, these small defects could be eliminated through recrystallization and rearrangement [56–58,59]. The evidence of dislocations and defects in the contact areas of the crystals also suggested that the growth of ZnO NPs underwent a direct particle fusion process.

**Scheme 2:**
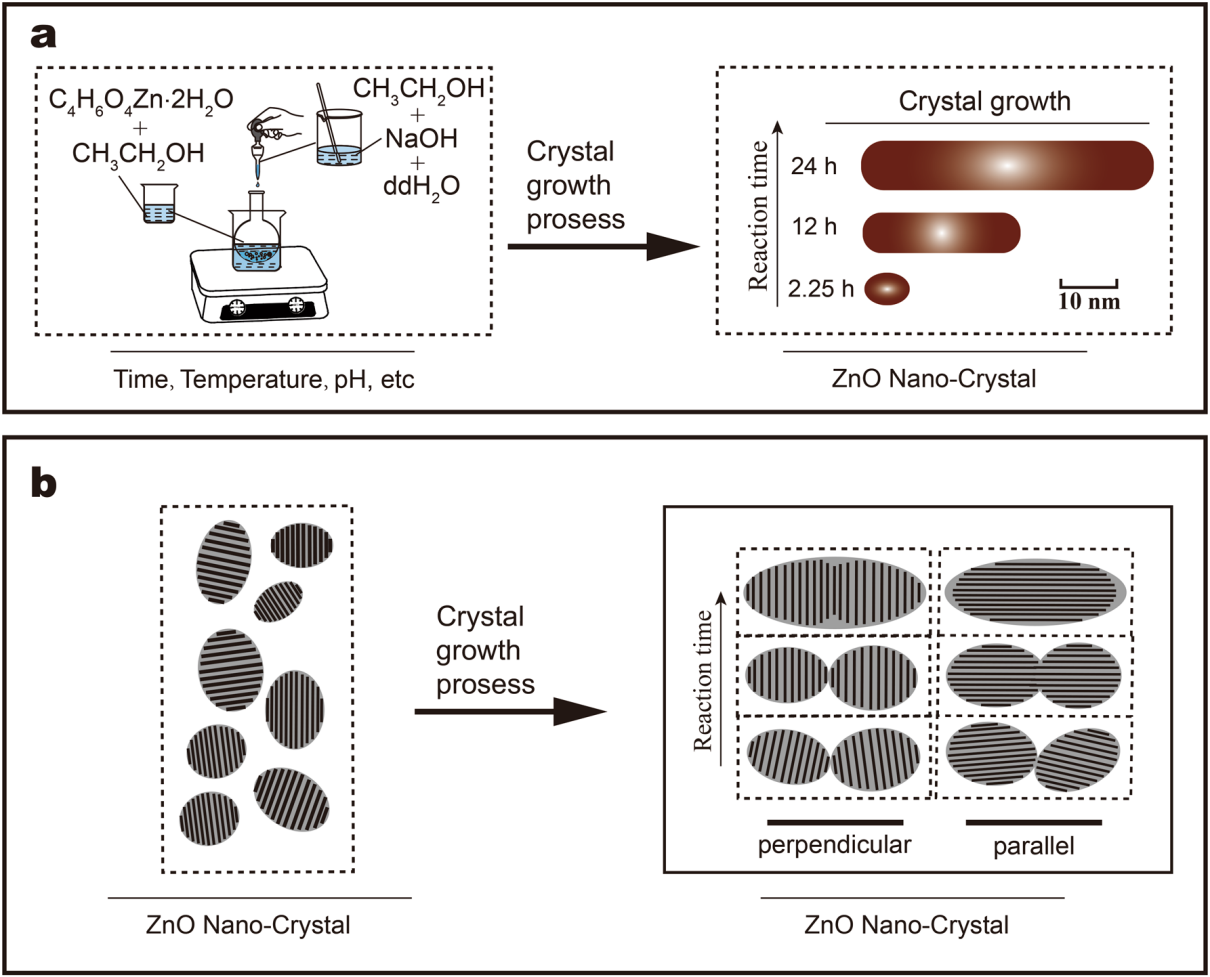
**a** The schematic illustration of the synthesis of ZnO NPs and the particle growth process. **b** The simulation of oriented attachment (OA) process of ZnO NPs growth

Based on the main points of the discussion above, we can conclude that the crystal growth of ZnO NPs conforms to the characteristics of the non-classical growth model. Furthermore, the mechanism for the growth process of ZnO NPs crystal was simulated as shown in Scheme 2b.

### FTIR analysis

We verified the formation of lipid shells at the surface of the ZnO nanoparticle, and the characteristic interactions between the lipid molecule and ZnO surfaces were also investigated using FTIR spectroscopy.

**Fig. 4:**
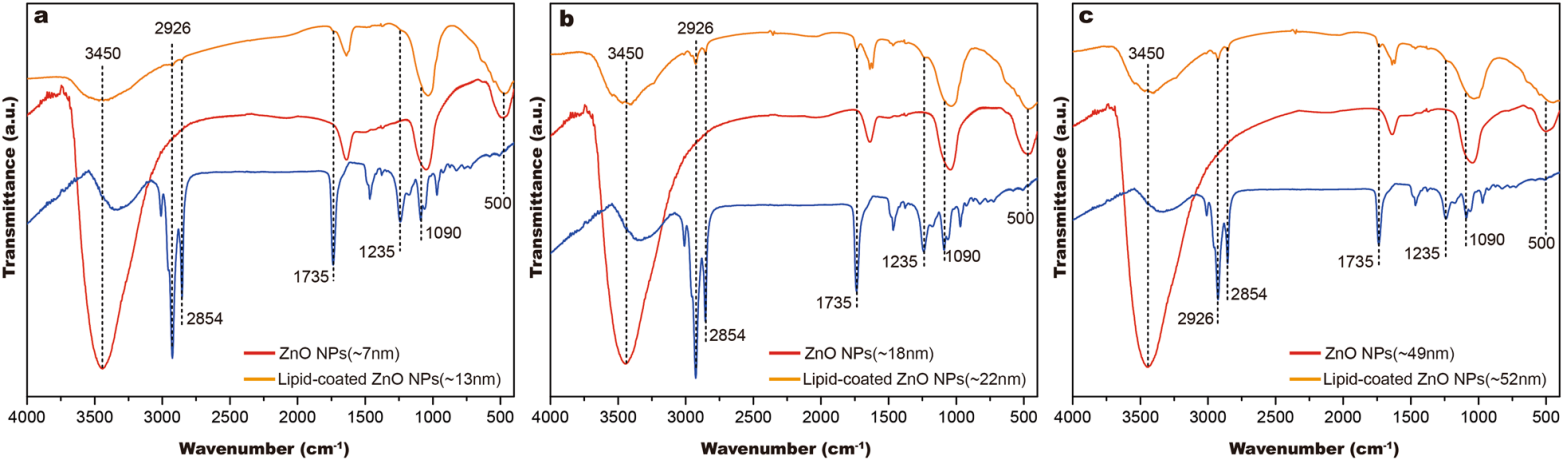
The structural properties of ZnO and lipid-coated ZnO NPs. The FT-IR spectra of **a** ZnO NPs(~ 7 nm) and lipid-coated ZnO NPs(~ 13 nm); **b** ZnO NPs(~ 18 nm) and lipid-coated ZnO NPs(~ 22 nm); **c** ZnO NPs(~ 49 nm) and lipid-coated ZnO NPs(~ 52 nm). The blue line represents the lecithin molecule

In the FT-IR spectrum (Fig. 4), the pristine and lipid-coated ZnO NPs displayed some common features. A typical Zn–O intense vibration was observed at around 440 cm^−1^. In the FT-IR spectrum of lipid-coated ZnO NPs sample, some intense peaks appeared at 2860, 2925 and 1750 cm^−1^, corresponding to the stretching vibration of –CH_x_ and C=O groups in the hydrophobic tail of the phospholipid, which confirmed the formation of the phospholipid layer on the surface of pristine ZnO NPs. The band from 3600 to 3400 cm^−1^, shows the stretch vibration of hydroxyl on the surface of pristine ZnO, in the lipid-coated ZnO NPs samples, their bands less are pronounced than that of the pristine ZnO NPs, which indicates the presence of the phospholipid layer on the surface of ZnO NPs.

Moreover, new stretching vibrations of P=O and P–O appeared at 1100 and 1235 cm^−1^ in the lipid-coated ZnO NPs sample. The disappeared peaks near 1250 cm^−1^ from the spectra of lipid-coated ZnO NPs implied that the lipid molecules chemically interacted with ZnO. Furthermore, the -OH band from 3600 to 3200 cm^−1^ and the Zn–O bond vibration peak were attenuated at 440 cm^−1^ (Fig. 4a–c). All these properties demonstrated the successful interactions between ZnO and lipid bilayer.

Unlike lipid-coated ZnO NPs, a broad band was observed from 3600 to 3200 cm^−1^ in the pristine ZnO NPs sample due to the stretching vibration of hydroxyl groups on the surface of the samples.

These results demonstrated that lipid shells were successfully formed on the ZnO nanoparticle surface.

### XRD analysis

**Fig. 5:**
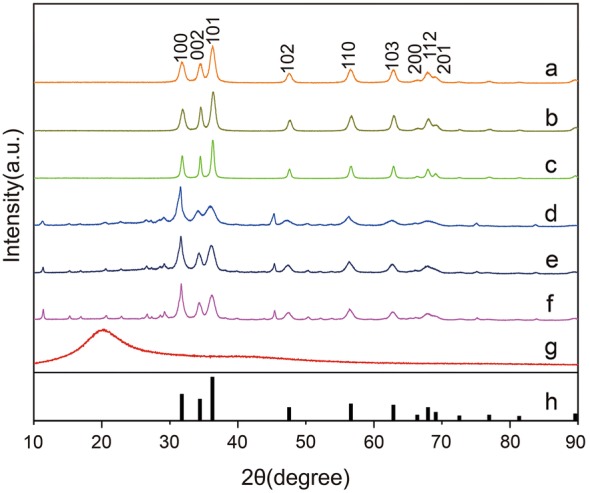
The XRD patterns of ZnO and lipid-coated ZnO NPs of different samples. **a** ZnO NPs(~7 nm); **b** ZnO NPs(~18 nm); **c** ZnO NPs(~49 nm); **d** Lipid-coated ZnO NPs(~13 nm); **e** Lipid-coated ZnO NPs(~22 nm); **f** Lipid-coated ZnO NPs(~52 nm). **g** Lecithin. **h** XRD pattern of bulk ZnO (according to JCPDS no. 36-1451) is shown at the bottom of each set of XRD patterns

Furthermore, the XRD results were compared with three shapes of pristine (Fig. 5a–c), lipid-coated ZnO NPs (Fig. 5d–f) and lecithin (Fig. 5g). The X-ray diffraction pattern shows that all samples have a hexagonal wurtzite crystal structure, which is consistent with the JCPDS card No. 36-1451 (Fig. 5h). No other phases (e.g. sphalerite) were observed. The average nanoparticle size (pristine ZnO NPs ~ 7 nm; analyzed using the Scherrer equation) was ~ 8 nm. This value was consistent with TEM results (Fig. 1a). The C-lattice constant, calculated from the XRD peaks of ZnO NPs (~ 7 nm) and ZnO NPs (~ 18 nm), was 0.26 nm. All patterns had broadened reflections due to the small particle sizes. These results are consistent with those obtained from TEM analysis.

Besides, a comparison of XRD patterns of samples at different reaction times demonstrated that the intensity of the (002) peak was enhanced from nanodots to nanorods (Fig. 5a–c), further indicating that ZnO NPs grew along the C-axis. However, after phospholipid encapsulation, the diffraction intensity decreased slightly (Fig. 5d–f). Compared to the original zinc oxide, the new diffraction peak appeared in the phospholipid encapsulated samples, which was consistent with the phospholipid spectrum (Fig. 5g). This outcome also supported our conclusion that the phospholipid bilayer successfully covered the surface of the nanocrystals.

### TG analysis

**Fig. 6:**
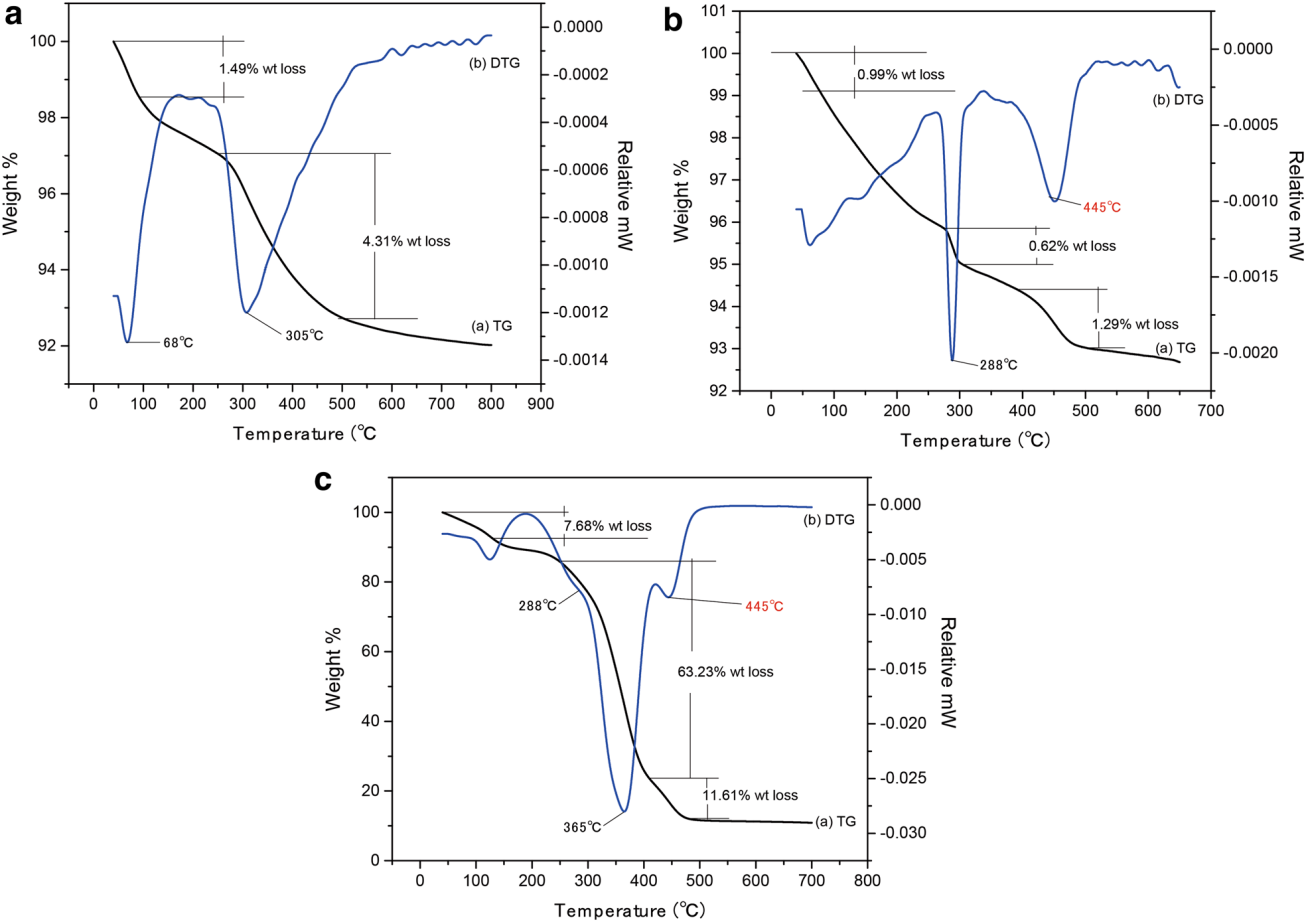
The TGA-DTG curves of pristine ZnO NPs ~ 7 nm. **a** Lipid-coated ZnO NPs ~ 13 nm **b** and **c** Lecithin

Figure 6 shows the results of TG analysis of pristine ZnO NPs (~ 7 nm), lipid-coated ZnO NPs(~ 13 nm), and lecithin. In Fig. 6a (Pristine ZnO NPs ~ 7 nm), two weight loss peaks could be observed. The mass fraction of bound water was about 1.49%, and the total weight loss of this sample was less than 8%, indicating the high purity of zinc oxide [60, 61]. In the curve of the lipid-coated ZnO NPs ~ 13 nm (Fig. 6b), three weight loss peaks could be observed. The first weight loss was observed due to the evaporation of bound water. The second and third peaks appeared due to the loss of lipid in the surface of ZnO nanoparticles. As the decomposition peak of phospholipid (Fig. 6c) at about 300 °C overlapped partially with the second peak of pristine ZnO NPs (~ 7 nm), it could not be observed obviously, but another decomposition characteristic peak of phospholipid could be observed in Fig. 6b at 445 °C. The peak around 445 °C corresponded to the decomposition of lecithin (Fig. 6c). The characteristic curve of phospholipid decomposition is shown in Fig. 6c. The decomposition temperature was between 200 °C and 450 °C.

For further investigating the condition of weight loss, the DTG of pristine ZnO NPs (~ 7 nm), lipid-coated ZnO NPs(~ 13 nm), and lecithin were studied. In Fig. 6, lipid-coated ZnO NPs(~ 13 nm) and lecithin displayed the same characteristic decomposition peak temperature at 445 °C. These results confirmed that the phospholipid molecule was successfully coated on the surface of ZnO NPs. Also, Additional file 1: Figure S2 shows the results of TG analysis of pristine ZnO NPs (~ 18 nm; ~ 49 nm) and lipid-coated ZnO NPs (~ 22 nm; ~ 52 nm).

### DLS analysis

The dispersion and agglomeration properties of NPs play important roles in the nanomaterial toxicology [62]. According to many reports, agglomeration causes a reduction in the specific surface area and activity. In biological applications, the agglomeration of drugs is one of the factors affecting their efficacy [63]. Many studies have proved that nanoparticles with good dispersion property show better therapeutic efficacy (antibacterial and bacteriostasis) or industrial catalytic property than the agglomerated particles [64].

**Fig. 7:**
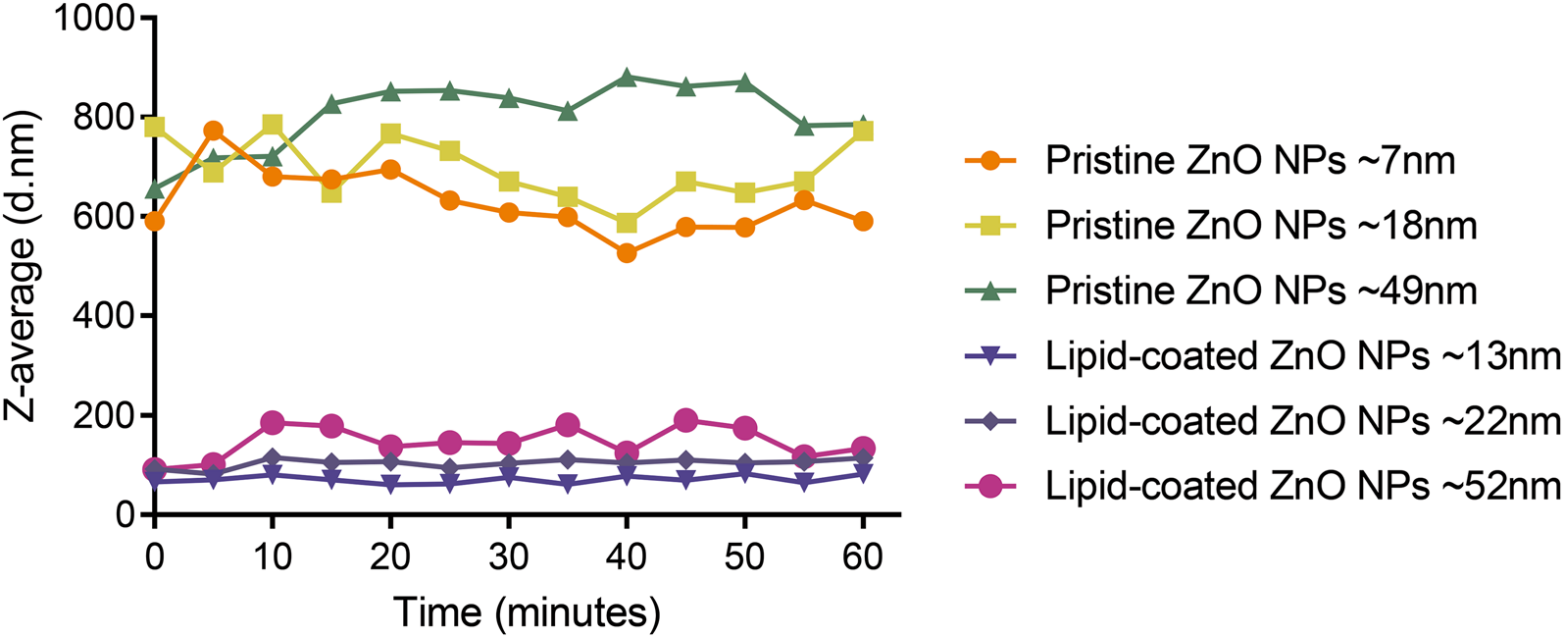
The DLS analysis of pristine ZnO NPs and lipid-coated ZnO NPs in terms of mean hydrodynamic size (Z-average) in water solution from 0 to 60 min

The colloidal stability and behavior of the pristine and lipid-coated ZnO NPs with different morphologies were characterized by DLS analysis in aqueous solution (Fig. 7). In aqueous solutions (rather than cell cultures), interference from other elements can be excluded. The above samples were exposed to liquid solvents, and the DLS behaviors versus time were recorded, which also represented the kinetics of pristine ZnO NPs and lipid-coated ZnO NPs in living systems to some extent. Particularly, as the lipid bilayer monomer was dissolved in alcohol solution, the DLS measurement of lipid-coated ZnO NPs was also performed in aqueous solution.

For determining the function of colloidal stability versus time, Z-average values were recorded in real-time in the aqueous solution. We tried to record the DLS analysis in water for more than 2 days. However, as the pristine ZnO NPs precipitated, the measurement could not meet the quality criteria. For consistency, we selected 0–60 min as the measurement period.

In Fig. 7, the average hydrodynamic diameter (Z-average) of the pristine ZnO NPs is significantly higher than that of the lipid-coated ZnO NPs (526 nm to 881 nm); so, pristine ZnO NPs tend to aggregate in aqueous solution. We also noticed that the pristine ZnO NPs without lipid-coating quickly form white fluffy precipitates. Meanwhile, the aggregation of lipid-coated ZnO NPs with different morphologies was also recorded. By contrast, the lipid-coated ZnO NPs samples exhibited a relatively small z-average (from 61 nm to 134 nm) in water; the sample demonstrated remarkable stability and dispersity. For a brief period, the pristine ZnO NPs remained suspended despite slight aggregation. We observed that all the samples were well dispersed in water. However, whether or not the ZnO NPs were coated with the lipid bilayer, the nanospheres always showed less aggregation compared to the nanorods.

Overall, in the aqueous solution, the pristine ZnO NPs showed a stronger aggregation than the lipid-coated ZnO NPs from the DLS level. According to published reports, the good dispersion and stability of lipid-coated ZnO NPs can be attributed to the fact that ZnO nanocrystal was coated with a phospholipid layer. The lipid coating prevented the contact of the ZnO nanocrystal surface to the aqueous solution, which may have resulted in their colloidally stable behavior.

### ICP analysis

The DLS results showed that different shapes of ZnO nanocrystal could significantly enhance the colloidal stability through lipid coating in aqueous solution. Further, we used ICP-OES to monitor zinc and phosphorus elements and analyzed the elemental dissolution of pristine and lipid-coated ZnO NPs in the water at each biostability time point (2, 12, 24, 48, 96 h).

**Fig. 8:**
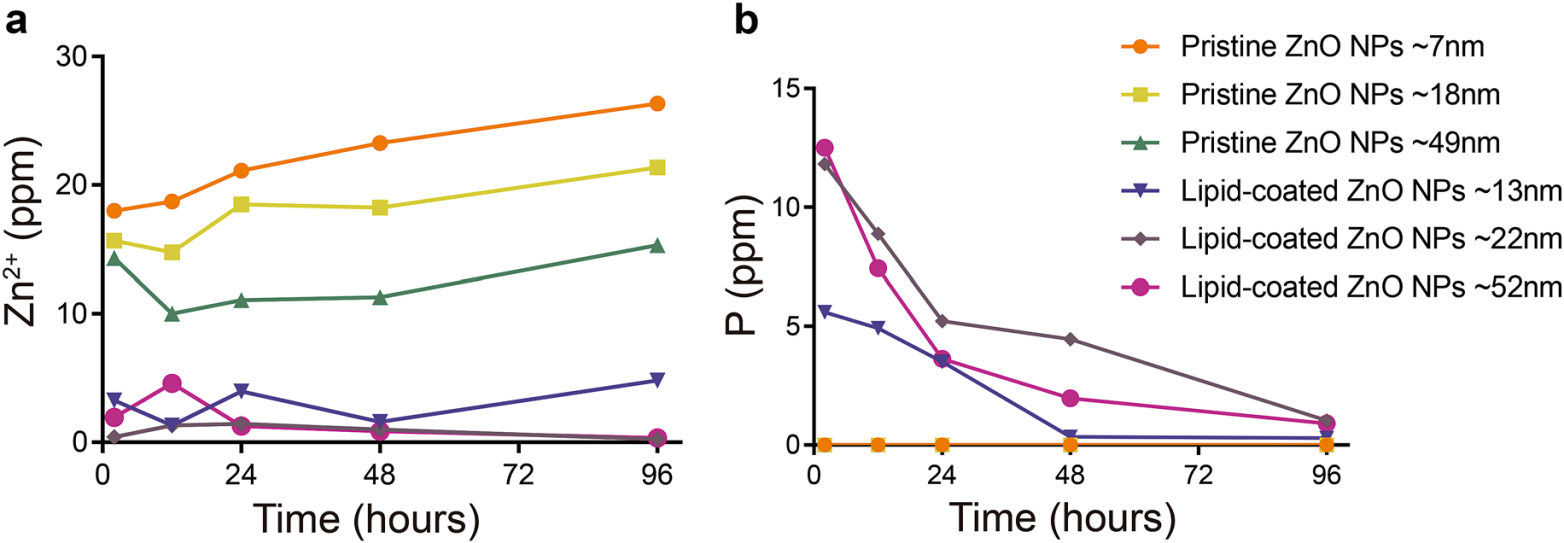
The ICP-OES analysis of **a** zinc and **b** phosphorus elements present in water at different time points of the biostability assays from different samples

In the pristine ZnO NPs, Zn^2+^ cations increased significantly from 17 to 26 ppm, 15 to 22 ppm and 10 to15 ppm (Fig. 8, pristine ZnO NPs ~ 7 nm, ~ 18 nm, ~ 49 nm). Throughout the experiment, the release of Zn^2+^ cations was significantly decreased with the increase in particle size (7 nm to 49 nm) of the pristine ZnO NPs.

Particularly, no significant change was observed in the composition of Zn^2+^ cation content in aqueous solution when lipid-coated ZnO NPs were exposed to water (only a slight change), and the presence of Zn^2+^ was almost undetectable (range from 0.23 ppm to 4.82 ppm). This evidence confirmed that the lipid-coated ZnO NPs samples were neither hydrolyzed nor dissolved.

A high degree of interaction has been reported between the phosphate anions and ZnO NPs. In our experiment, the number of phosphorus elements slowly decreased (from 12 ppm to around 1 ppm) with time (Fig. 8b), indicating the possible precipitation of phosphate groups on this sample surface. In this study, the phosphorus in the solution might have reacted with the NPs, and carbonate and phosphate groups were precipitated on ZnO NPs. The precise conclusion needs to be supported by further experimental results.

These data indicate a slight dissolution of these samples and prove the sustained-release properties of the phospholipid layer. In conclusion, the phospholipid bilayer self-assembled on the surface of inorganic nanocrystals to form a complete and dense covering layer thus prevented the reaction between ZnO nanocrystal and the aqueous solution. As a lipid molecule contain a hydrophilic head and a hydrophobic tail, it can protect the ZnO NPs from hydrolysis and dissolution, thus rendering sustained-release effects. Pujalte et al. reported that CdS NPs could induce oxidative stress and cytotoxicity by releasing Cd^2+^ in cells [65]. Also, we assume that the ZnO NPs release many Zn^2+^ at high concentrations, induce ROS production, which in turn reduces the cellular defenses, destroy the cell’s DNA and other structures, and ultimately cause cell death. Besides, the high toxicity of ZnO NPs is related to the mass level of Zn^2+^ in the solution [66]. ZnO causes cytotoxicity by releasing Zn^2+^ cation and producing oxidative stress. Zn^2+^ cation can be released from pristine ZnO NPs and lipid-coated ZnO NPs, which would induce inflammatory responses and result in cellular damage, cytotoxicity, and apoptosis [67].

Alteration of Zn^2+^ cation content in aqueous solution is indicative of oxidative stress [68]. Even though zinc is an essential metal, excess Zn^2+^ ion can cause inflammatory reactions in the body. The ICP data displayed the dissolution status of ZnO NPs in aqueous solution, and the relationship between Zn^2+^ ion and cytotoxicity was verified by these results.

### Cytotoxicity assay

Many studies have been conducted for determining the toxicity of ZnO NPs, but the mechanism of cytotoxicity is still unclear. Many parameters affect cytotoxicities, such as particle size, morphology, and surface chemical composition, treatment time, intake rate, and drug concentration [69]. We studied the difference in cytotoxicity mechanism and particle intake of ZnO and lipid-coated ZnO with different morphologies and concentration, by conducting cytotoxicity and apoptosis assay on HeLa cells using these samples. Furthermore, the morphological changes in cell-nucleus were characterized by biological transmission electron microscopy (BTEM) after exposing them to the samples.

HeLa cells are extremely suitable for studying cytotoxicity. In this experiment, the concentrations of the samples were varied systematically from 15 to 200 μg/mL. The CCK-8 assays were performed using these sample concentrations on the HeLa cell culture for 24 h, and the proliferation of HeLa cells was evaluated. By comparing the cytotoxicity of pristine ZnO NPs (~ 7 nm, ~ 18 nm, ~ 49 nm) and lipid-coated ZnO NPs (~ 13 nm, ~ 22 nm, ~ 52 nm) with different morphologies, the effects of morphology and lipid coating on cytotoxicity were evaluated. We freshly prepared all the samples in this experiment for reducing the aggregation of the nanomaterials.

**Fig. 9:**
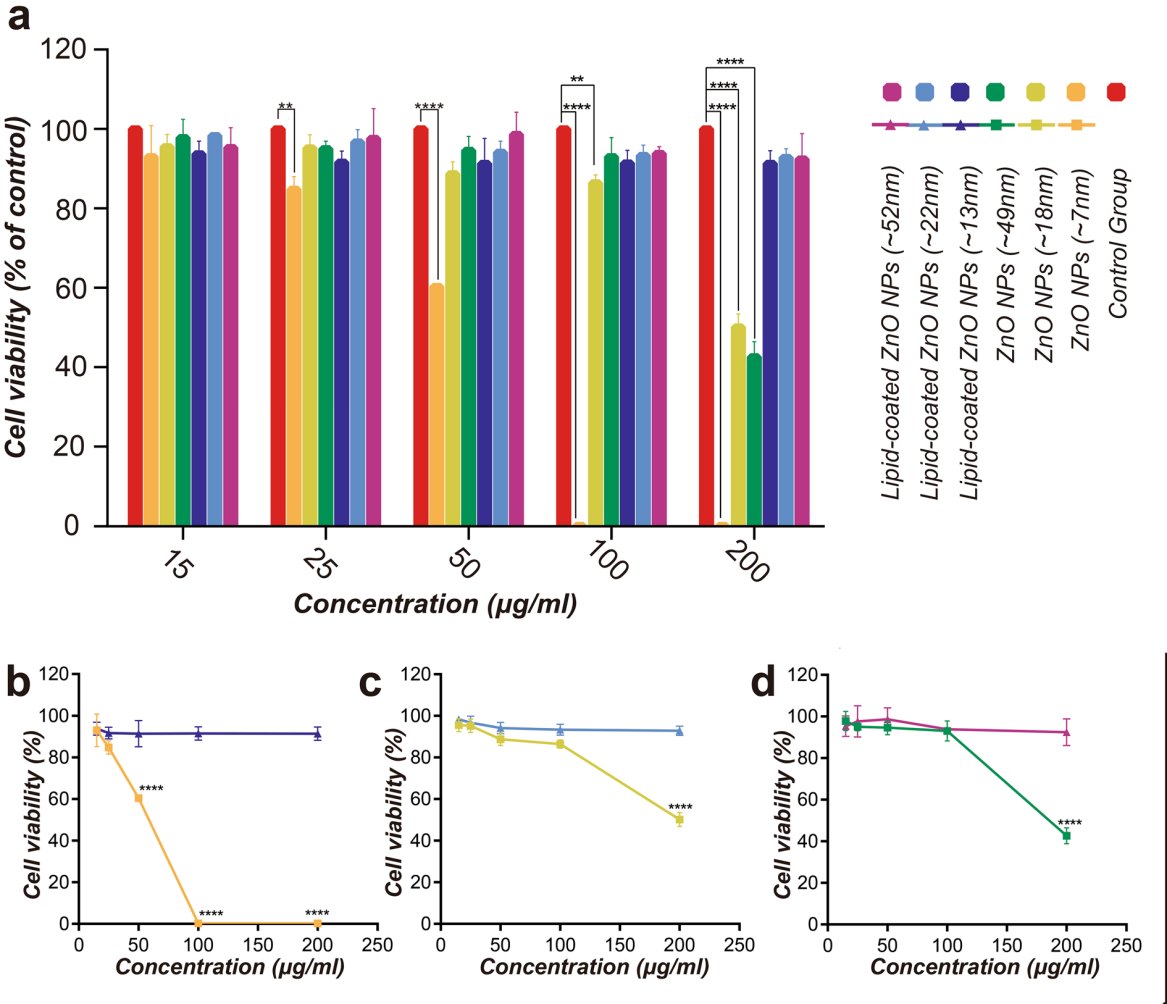
The cell viability of HeLa cells after 24 h of exposure to ZnO and lipid-coated ZnO NPs. **a** Control group vs ZnO NPs (~ 7 nm; ~ 18 nm; ~ 49 nm) and lipid-coated ZnO NPs (~ 13 nm; ~ 22 nm; ~ 52 nm); **b** ZnO NPs (~ 7 nm) vs lipid-coated ZnO NPs (~ 13 nm); **c** ZnO NPs (~ 18 nm) vs lipid-coated ZnO NPs (~ 22 nm); **d** ZnO NPs (~ 49 nm) vs lipid-coated ZnO NPs (~ 52 nm). The activity was measured with CCK-8 assay, and the data were expressed as mean ± SD (standard deviation) of three experiments. *p < 0.05, **p < 0.01, ***p < 0.001, ****p < 0.0001

According to Fig. 9, the cytotoxicities of these samples were different. When the concentration was 15 μg/mL, no sample exhibited any cytotoxicity. At 200 μg/mL, all ZnO NPs (~ 7 nm, ~ 18 nm, ~ 49 nm) produced significant cytotoxic effects. In contrast, no significant inhibition on cell growth was observed after 24-h of exposure of lipid-coated ZnO NPs (~ 13 nm, ~ 22 nm, ~ 52 nm) at 15, 25, 50, 100 and 200 μg/mL (p > 0.05). The cell viability of pristine ZnO NPs with diverse morphologies (~ 7 nm, ~ 18 nm, ~ 49 nm) was significantly different at the same concentration level. The pristine ZnO NP of ~ 7 nm particle size was very toxic, even at a low concentration (25 μg/mL). For example, exposure to the ZnO NPs of ~ 18 nm and 49 nm particle sizes demonstrated the cell viability of 0.89 ± 0.03 and 0.95 ± 0.03, respectively at 50 μg/mL; when the concentration was increased to 200 μg/mL, the cell viability was decreased to 0.5 ± 0.03 and 0.43 ± 0.04, respectively. This result also indicated that ZnO NPs (~ 7 nm, ~ 18 nm, ~ 49 nm) exhibited significant dose and morphology-dependent toxicity against the HeLa cell. The pristine ZnO NPs were significantly more cytotoxic than the lipid-coated ZnO NPs, irrespective of morphology or concentration level (15 ~ 200 μg/mL) (Fig. 9). The minimum inhibitory concentrations (MIC) of ZnO NPs (~ 7 nm), ZnO NPs (~ 18 nm), ZnO NPs (~ 49 nm) were calculated to be 59.51, 239.7, and 201.2 μg/mL, respectively. We subsequently selected sample-concentrations at the MIC to further investigate the cellular uptake of nanoparticles and cellular apoptosis in the following experiments.

### Annexin V-FITC/propidium iodide (PI) apoptosis assay

Further, we analyzed the induction of apoptosis by the samples to HeLa cells after 24-h of incubation. Dual staining with annexin V-FITC and PI method was used to quantitatively determine the early apoptosis, late apoptosis, and necrosis of cells [70].

To explore the differences in apoptosis between ZnO NPs and lipid-coated ZnO NPs at the same concentration, we divided the test samples into three groups according to the particle morphology (ZnO ~ 7 nm and lipid-coated ZnO NPs ~ 13 nm, exposure at 40 μg/mL; ZnO ~ 18 nm and lipid-coated ZnO NPs ~ 22 nm, exposure at 120 μg/mL; ZnO ~ 49 nm and lipid-coated ZnO NPs ~ 52 nm, exposure at 150 μg/mL). The exposure concentration was determined by the MIC as described in the previous section.

**Fig. 10:**
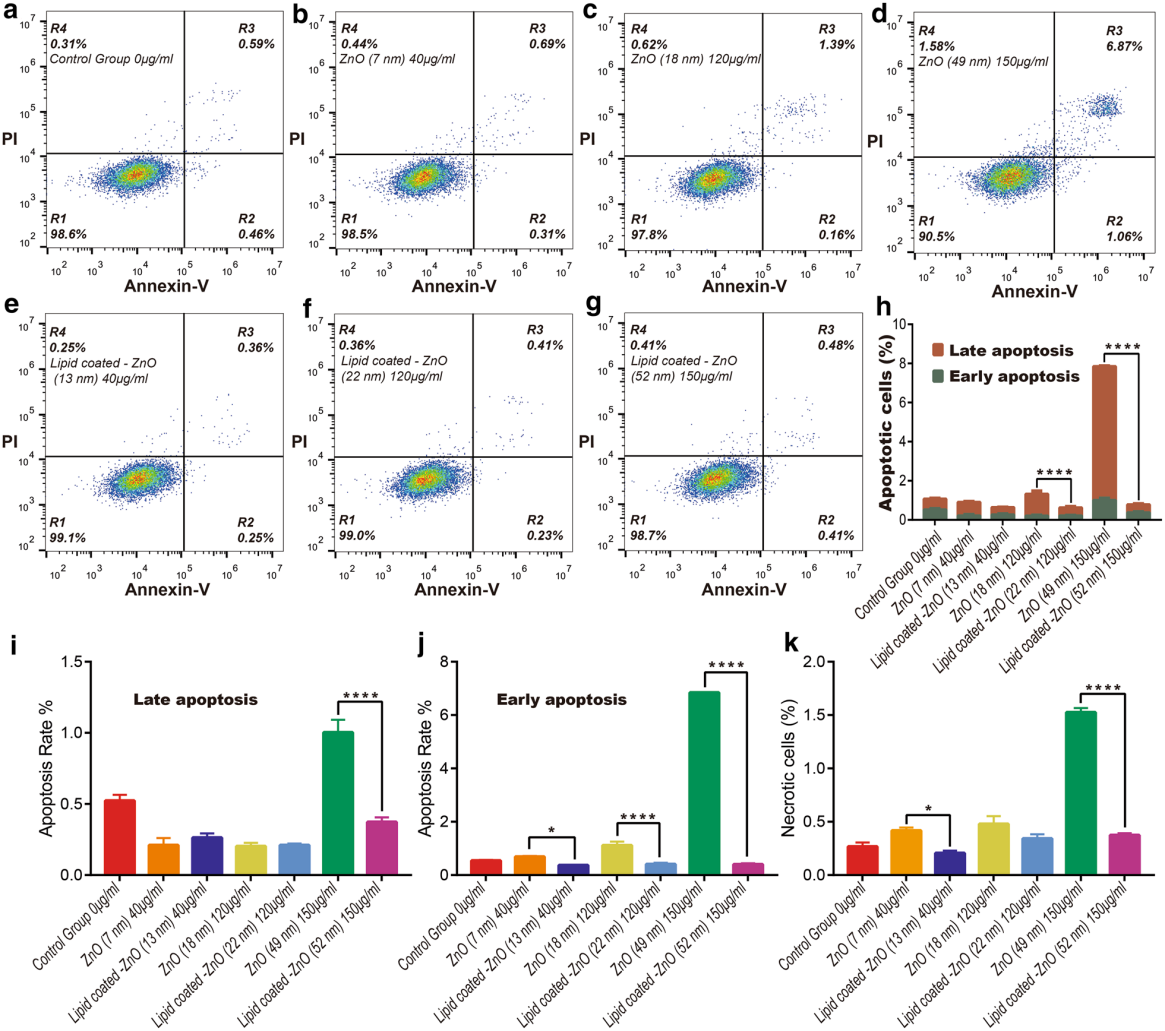
The apoptosis and necrosis of HeLa cells after 24-h of the exposure of ZnO NPs and lipid-coated ZnO NPs as detected by flow cytometry. **a**–**g** The representative flow cytometer images of HeLa cells after being exposed to pristine ZnO NPs (~ 7 nm; ~ 18 nm; ~ 49 nm) and lipid-coated ZnO NPs (~ 13 nm; ~ 22 nm; ~ 52 nm) for 24 h (0, 40, 120 and 150 μg/mL). **h**–**k** The quantitative results of apoptotic and necrotic percentages from flow cytometry analysis. *p < 0.05, **p < 0.01, ***p < 0.001, ****p < 0.0001

Similar to the cytotoxicity, apoptosis was also concentration-dependent. In Fig. 10a–g R1, R2, R3, and R4 represent viability, early apoptosis, late apoptosis, and necrotic HeLa cells, respectively.

After 24-h of exposure, apoptosis increased significantly for ZnO ~ 18 nm (exposure at 120 μg/mL) and ZnO ~ 49 nm (at 150 μg/mL) compared to their lipid-coated counterparts (lipid-coated ZnO NPs ~ 22 nm exposure at 120 μg/mL and lipid-coated ZnO NPs ~ 52 nm exposure at 150 μg/mL). The proportions of apoptotic cells of ZnO ~ 49 nm and lipid-coated ZnO NPs ~ 52 nm at 150 μg/mL were 7.93% and 0.66%, respectively.

In Fig. 10h, the ZnO ~ 49 nm (exposure at 150 μg/ml) exhibited the highest rate of early and late apoptosis (1.00 ± 0.15% and 6.84 ± 0.03%). In the rest of the group, the apoptosis rate was below 2% and exhibited no differences from the control group. In the 120 μg/mL group, the rate of apoptosis of lipid-coated ZnO NPs ~ 22 nm was significantly lower than that of ZnO ~ 18 nm. These values significantly demonstrated that phospholipid encapsulation could reduce the rate of apoptosis in the high dose. Meanwhile, this result was also depicted in R2, R3, and R4 (Fig. 10i–k). ZnO ~ 7 nm and ZnO ~ 18 nm groups exhibited overt cytotoxicity to HeLa cells (MIC, 37.51 μg/mL and 108.32 μg/mL) compare to the control group, but induced a significant decrease in cellular apoptosis.

Besides, necrosis was observed in less than 2% of cells across all the groups (Fig. 10k), indicating that under the treatment of our sample, the pathway of HeLa cell necrosis was primarily due to apoptosis. At the same concentration, the late apoptosis rate of lipid-coated ZnO NPs (~ 13 nm; ~ 22 nm; ~ 52 nm) group was always lower than that of ZnO NPs (~ 7 nm; ~ 18 nm; ~ 49 nm) group (Fig. 10i). Two main reasons for this phenomenon were: 1. Due to the coating of phospholipid on ZnO NPs, the release of zinc ions was slowed down, thus reducing the cytotoxicity. 2. Phospholipids and cholesterol contributed to the reproduction of cells and enhanced ion tolerance of cells. Further evidence is needed to verify the second point.

### The BTEM analysis

**Fig. 11:**
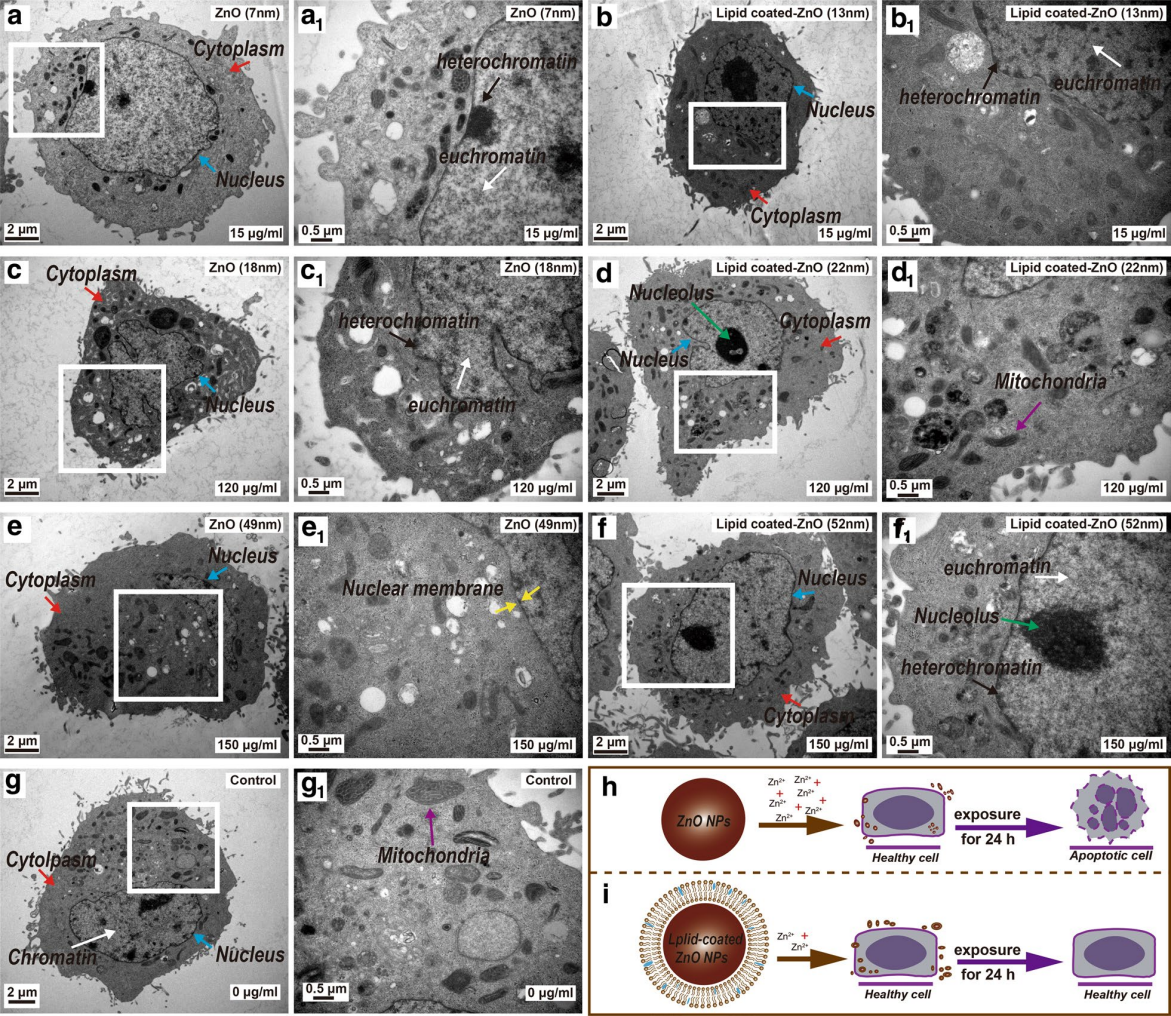
The TEM images of the nucleus, nucleolus, cytoplasm, and the nuclear membrane morphology after being exposed to pristine ZnO NPs and lipid-coated ZnO NPs (0, 15, 120, and 150 μg/mL) for 24 h. The images in Panel (**a**_**1**_–**g**_**1**_) are enlarged from the panel (**a**–**g**). The blue arrows indicate the malformation of nuclear morphology after NPs exposure (nuclear membrane rupture). The black and white arrows indicate the chromatin condensation (electron-dense, black structure along nuclear membrane) within the nuclei. The images in panel (**h**–**i**) are simulated exposure process of NPs

The morphological changes of the HeLa cell nucleus were characterized using TEM (Fig. 11) after sample treatment. Figure 11g shows the TEM images of the control group (0 μg/mL). Lipid-coated ZnO NPs group (Fig. 11b, d, f) and pristine ZnO NPs (7 nm, 15 μg/mL) group (Fig. 11a) exhibited intact nuclear membrane, complete mitochondrial structure, randomly distributed chromatin and clear organelle structure without obvious modification. Pristine ZnO NPs (18 nm, 120 μg/mL) group displayed cellular apoptosis (Fig. 11c), including shrinking of the inner nuclear envelope, coiled or congealed and marginalized cell nucleus chromatin or long-chain structures (black arrows in Fig. 11), and deformed and vacuolar mitochondria. The pristine ZnO NPs (49 nm, 150 μg/mL) group exhibited typical phenomena of apoptosis (Fig. 11e), where the nuclei were under obvious pyknosis into a heterogeneous block structure, with small apoptotic bodies; nuclear morphology became more irregular and malformed or blurred or vanished mitochondrial cristae. The images in Fig. 11h, i exhibit the simulated exposure process of NPs.

Apoptosis is characterized by the loss of nuclear membrane integrity and chromatins condensation. NPs treatment causes morphological changes and loss of function in the cells. Our experiment displayed that compared to lipid-coated ZnO NPs, the pristine ZnO NPs significantly more affected the nuclear morphology.

The literature has shown that endocytosis of nanomaterials by cells is generally below 300 nm, and endocytosis is inefficient if the nanomaterials are too large or too small. In aqueous solutions, 50–60 nm is most likely to be endocytosed within a relatively short period. Also, nanoparticle could be transferred across the cell membrane and distributed to the cytoplasmic areas [71].

HeLa cells were observed after being exposed to different samples (exposure concentration and sample grouping were the same as apoptosis tests). Interestingly, after 24 h of exposure, we did not observe NPs entering cells, but HeLa cells still showed evidence of apoptosis, because the sample concentration was low and the exposure time was too short, so the NPs could not enter the cell, and they were only adsorbed on the cell surface in small amounts. This local concentration effect may lead to the indirect functional loss of organelles and be responsible for the cytotoxicity of nanoparticles. Thus, we hypothesize that this local concentration effect and oxidative stress generated by NPs are the major causative factors of HeLa cell damage. This result also confirmed that the toxicity of ZnO NPs was not produced due to the direct cellular uptake of the particles. Further research is needed for verifying this hypothesis. Meanwhile, compared to ICP data, the release of zinc ions more significantly influenced the cytotoxicity.

Based on the above evidence, we can conclude that the phospholipid bilayer can self-assemble on the surface of inorganic nanocrystals to form a complete and dense covering layer, thus prevent the reaction between ZnO nanocrystal and the aqueous solution. The hydrophobic tail of the phospholipid:

1. Promotes good dispersion of the ZnO NPs as a colloidal suspension in aqueous solution.
2. Prevents the dissolution of ZnO NPs and maintains their physical and chemical properties.
3. The phospholipid layer decelerates the release of Zn^2+^ cation and plays an important role in the sustained release.

Based on the above features, lipid-coated ZnO NPs are more likely to reduce the cytotoxicity.

## Conclusions

In this study, we synthesized ZnO with different morphologies (pristine ZnO NPs ~ 7 nm, ~ 18 nm, ~ 49 nm) based on OA (oriented attachment) model, and demonstrated that the ZnO NPs surface could successfully self-assemble the phospholipid bilayer and improve the colloidal stability, prevent the aggregation and dissolution of nanocrystal particles in the solution, and avoid the dissolution of ZnO nanomaterials into Zn^2+^ cations, thus, reduce cytotoxicity and stress response. The surface chemistry of these samples influences their biostability in aqueous solution. We recorded the differences in the aggregation or dissolution behavior of different samples in aqueous solutions and explained these results. Our results showed that compared to the pristine ZnO NPs (~ 7 nm; ~ 18 nm; ~ 49 nm), lipid-coated ZnO NPs (~ 13 nm; ~ 22 nm; ~ 52 nm) could improve the colloidal stability, prevent the aggregation and dissolution of nanocrystal particles in the solution, and avoid ZnO NPs dissolution into Zn^2+^ cations, and thus reduce their cytotoxicity. Compared to the lipid-coated ZnO NPs group, pristine ZnO NPs (~ 7 nm; ~ 18 nm; ~ 49 nm) damaged the cells at lower concentrations, in a dose-dependent manner. At the same concentration, ZnO NPs (~ 7 nm) exhibited the highest cytotoxicity. Noteworthily, for lipid-coated ZnO NPs, the higher level of concentration doesn’t suggest higher cytotoxicity.

Overall, the results mentioned above clearly revealed the physical and chemical properties of pristine and lipid-coated ZnO NPs nanocrystal with different morphologies and their behavior in biological media. This information is of great significance for increasing the drug efficacy, reducing the cytotoxicity and even developing the application basis of multi-functional inorganic nanomaterials.

## Supplementary information

**Additional file 1: Figure S1. a** The EDX spectra of lipid-coated ZnO NPs (~ 22 nm) and **b.** Lipid-coated ZnO NPs (~ 52 nm).

**Additional file 2: Figure S2.** The TGA-DTG curves of **a-b:** Pristine ZnO NPs ~ 18 nm and ~ 49 nm; **c-d:** Lipid-coated ZnO NPs ~ 22 nm and ~ 52 nm.

## Data Availability

The authors declare the availability of data and materials that were used for this study at Zhongkai University of Agriculture and Engineering.

## Abbreviations

NPs: Nanoparticles
TEM: Transmission electron microscopy
HR-TEM: High-resolution transmission electron microscopy
EDX: Energy Dispersive X-Ray Spectroscopy
SAED: Selected area electron diffraction
OA: Oriented attachment
XRD: X-ray diffraction
DLS: Dynamic light scattering
FFT: Fast Fourier transform
FTIR: Fourier transform infrared
TGA: Thermogravimetric Analysis
ICP-OES: Inductively Coupled Plasma Optical Emission Spectrometry
CCK-8: Lower nucleation energy barrier
MIC: Minimum inhibitory concentrations
BTEM: Lower nucleation energy barrier
PI: Propidium iodide
Dh: Hydrodynamic diameter
