# Supplementary figures and images for: Lipid-coated ZnO nanoparticles synthesis, characterization and cytotoxicity studies in cancer cell
Source: Nano Converg. 2020 Apr 23;7:14. doi: 10.1186/s40580-020-00224-9 (PMC7181468; doi:10.1186/s40580-020-00224-9)

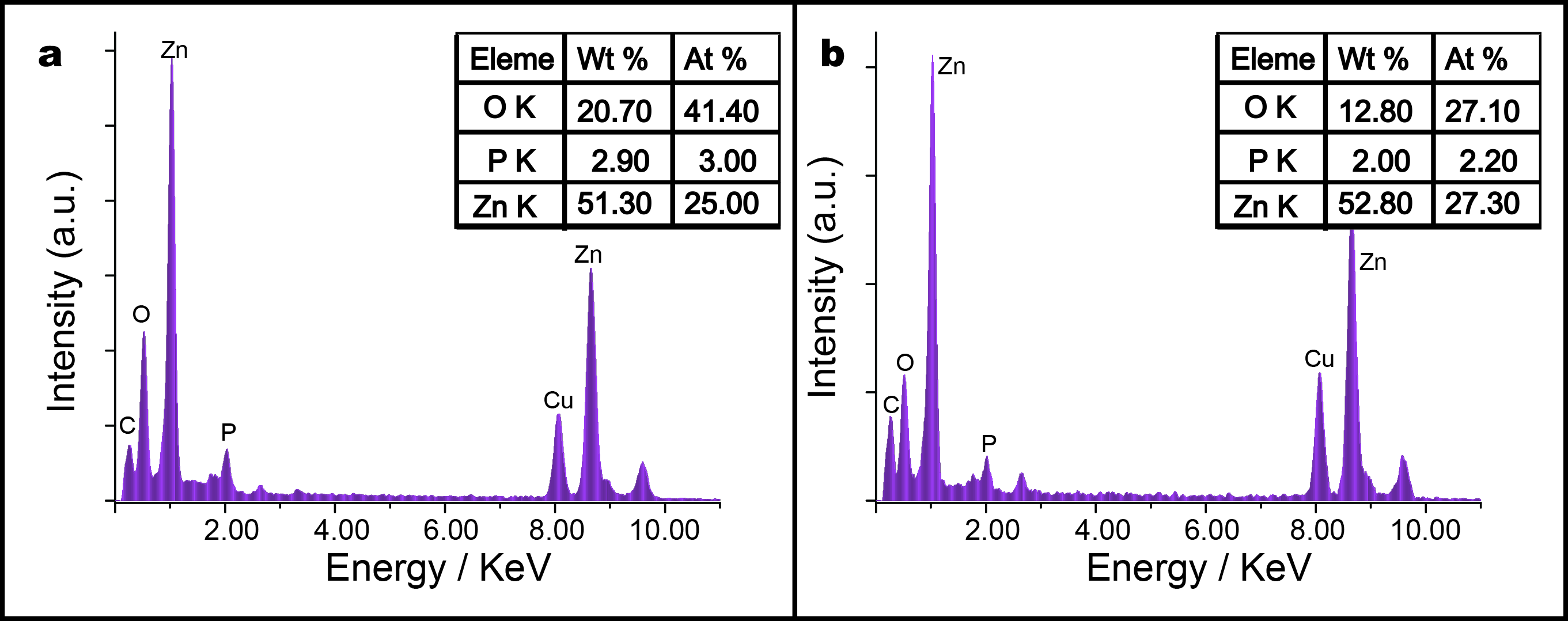

Supplement: Supplementary file 1 — Additional file 1: Figure S1. a The EDX spectra of lipid-coated ZnO NPs (~ 22 nm) and b. Lipid-coated ZnO NPs (~ 52 nm). [file 40580_2020_224_MOESM1_ESM.tif]

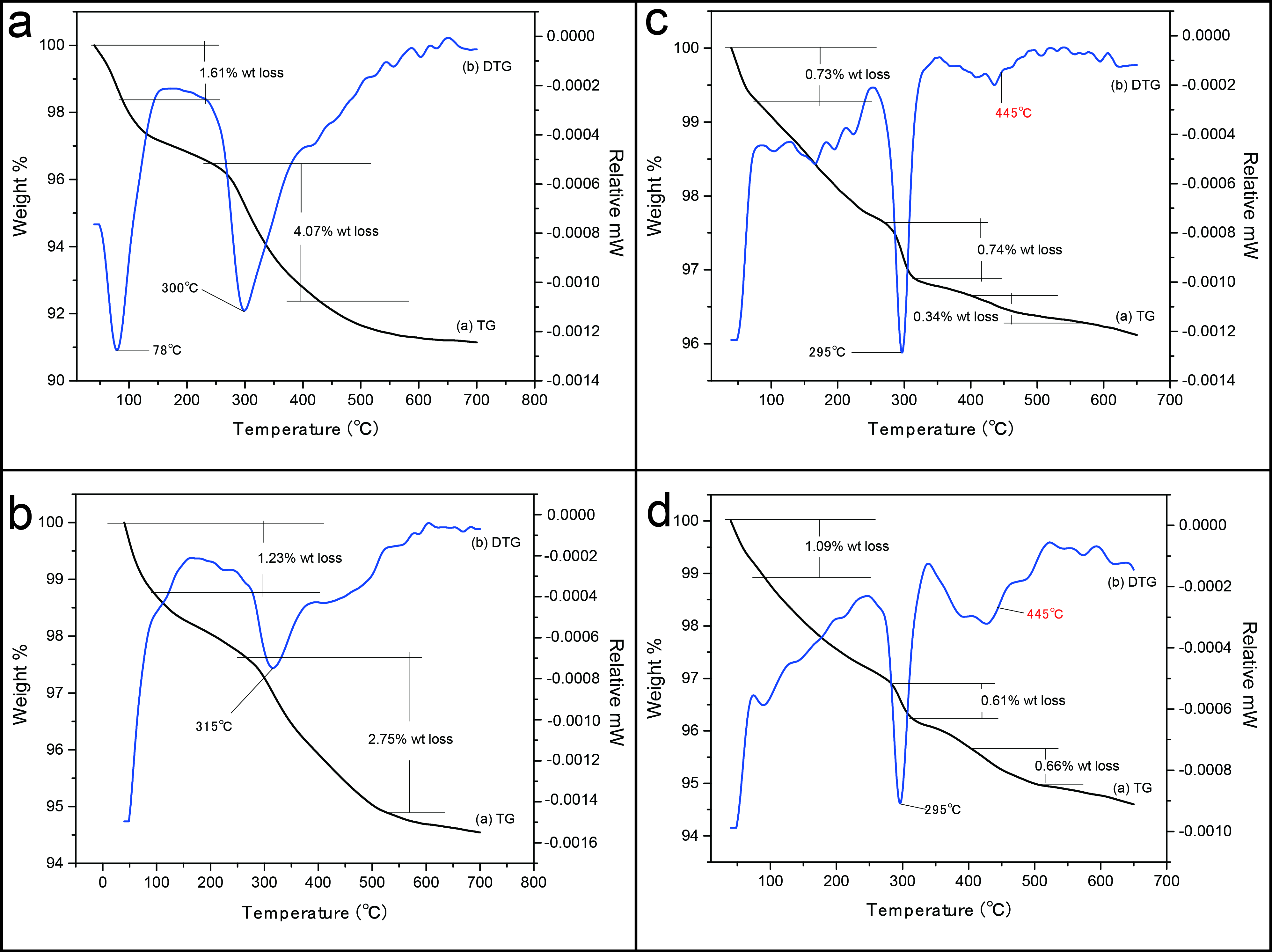

Supplement: Supplementary file 2 — Additional file 2: Figure S2. The TGA-DTG curves of a-b: Pristine ZnO NPs ~ 18 nm and ~ 49 nm; c-d: Lipid-coated ZnO NPs ~ 22 nm and ~ 52 nm. [file 40580_2020_224_MOESM2_ESM.tif]
